# Unveiling the immunomodulator role of plasma oxidized lipids in SA-AKI progression: a CRRT perspective

**DOI:** 10.3389/fphys.2024.1412235

**Published:** 2024-12-23

**Authors:** Lu Zhou, Huirong Li, Wenfeng Guo, Lan Feng, Jiangtao Hu, Jing Liu, Tingting Wang, Hongbao Liu

**Affiliations:** Department of Nephrology, Tangdu Hospital, The Fourth Military Medical University, Air Force Medical University, Xi’an, Shaanxi Province, China

**Keywords:** acute kidney injury, sepsis, oxidative lipidomics, prognosis, immune microenvironment, bioactive lipids

## Abstract

**Background:**

Plasma oxidized lipids are intimately linked to immune regulation as bioactive mediators. However, it is not clear whether they are related to the progression of sepsis-associated acute kidney injury (SA-AKI) and the effect of continuous renal replacement therapy (CRRT). This study intends to explore the changes in certain oxidized lipid during CRRT treatment and their correlation with the immune microenvironment and prognosis by analyzing plasma oxidative lipidomics.

**Methods:**

A total of 48 SA-AKI patients undergoing CRRT for more than 72 h were enrolled in this prospective cohort study. Oxidative lipidomics was analyzed by ultra performance liquid chromatography coupled with tandem mass spectrometric (UPLC-MS/MS) detection at the beginning of CRRT (T0) and 72 h later (T72), respectively.

**Results:**

Compared with survivors, plasma EETs, EpOMEs and EpDPEs in non-survivors were significantly down-regulated at T0, while PGFs, TXB and HEPEs were up-regulated. After 72 h of CRRT, DiHETEs were significantly up-regulated and PGFs were down-regulated in non-survivors, while HEPEs and EpOMEs were up-regulated and 6keto-PGF1α was down-regulated in survivors. KEGG annotation showed that the differential lipids of survivors before and after CRRT were mainly enriched and up-regulated in metabolic pathway.

**Conclusion:**

This study provided a comprehensive overview of plasma oxidized lipids in SA-AKI patients undergoing CRRT and further elucidated the lipids and pathways linked to patient severity and prognosis. Additionally, we unveiled the potential mechanisms by which CRRT improves the prognosis of SA-AKI patients by removing PGFs and TXs while simultaneously upregulating HEPE to ameliorate the immune microenvironment, as well as the potential significance of adjusting CRRT prescriptions based on plasma oxidized lipidomics.

## 1 Introduction

Acute kidney injury is a frequent complication in patients admitted to an intensive care unit (ICU) for sepsis and independently associated with mortality and a high level of resource use (Quenot et al., 2013; Hoste et al., 2015; Mehta et al., 2015). Continuous renal replacement therapy (CRRT) is the primary method for critical acute kidney injury (AKI), but due to the unique nature of sepsis, the response of sepsis-associated acute kidney injury (SA-AKI) to CRRT may differs from other types of AKI without sepsis (Dellepiane et al., 2016; Bellomo et al., 2017). Despite considerable research efforts, it is still unclear whether and when CRRT should be commence to improve outcome of patients with SA-AKI.

SA-AKI is primarily caused by the inflammatory cascade triggered by the release of a large number of inflammatory mediators during sepsis, as well as immune dysfunction, leading to microcirculatory failure, mitochondrial damage, and subsequent cellular and organ injury (Zarbock et al., 2023). As endogenous signaling molecules, plasma oxidized lipids are closely associated with the complex pathophysiological environment induced by sepsis through regulating pro-inflammatory and anti-inflammatory responses (Fullerton et al., 2014; Willenberg et al., 2016; Chiang et al., 2017; Chen et al., 2020; López-Vicario et al., 2020). Recent studies have identified a specific plasma oxidized lipid profile in SA-AKI patients, which is closely linked to the occurrence and progression of SA-AKI (Zhou et al., 2023). Overall, these studies reinforce the concept that cell/organ injury in SA-AKI is triggered by changes in the immune microenvironment mediated by oxidative lipids. However, there are limited data available on whether specific oxidized lipids can serve as reliable markers for assessing the severity and prognosis of SA-AKI patients undergoing CRRT and the impact of CRRT on it.

Given the complexity and diversity of lipid metabolism network, a new branch of lipidomics, oxidative lipidomics, appears and provides a powerful tool for studying the development of disease states (Lydic et al., 2015; To et al., 2015; Wang et al., 2016). This study used ultra performance liquid chromatography coupled with tandem mass spectrometric detection (UPLC‒MS/MS) combined with machine learning algorithm to analyze the alteration in certain oxidized lipid during CRRT treatment and their correlation with the immune microenvironment and prognosis in SA-AKI patients. Additionally, we aim to uncover the underlying mechanisms of CRRT in the treatment of SA-AKI through annotations from the Kyoto Encyclopedia of Genes and Genomes (KEGG), which can offer valuable insights into how CRRT impacts the molecular pathways involved in SA-AKI, and help optimize the use of CRRT in managing SA-AKI and potentially identify new therapeutic targets for improving outcomes in critical SA-AKI patients.

## 2 Materials and methods

This is a prospective observational cohort study conducted in the ICU of Tangdu Hospital from April to September in 2023. This study was approved by the Ethics Committee of Tangdu Hospital (K202302-08), registered on the www.chictr.org.cn (ChiCTR2300069667) and conducted in accordance with the Declaration of Helsinki. Results are reported by the guidelines according to the Strengthening the Reporting of Observational Studies in Epidemiology (STROBE). All participants or their legal representatives agreed to plasma collection and provided written informed consent.

### 2.1 Subjects

The eligible patients were all critical ill patients with SA-AKI who have been determined by the immobilized clinical team to commence CRRT. The diagnosis of sepsis was determined by the Third International Consensus Definitions for Sepsis and Septic Shock (Sepsis-3), while the diagnosis of AKI diagnosis was based on the Kidney Disease: Improving Global Outcomes (KDIGO) guidelines. Exclusion criteria were life expectancy <24 h, pregnancy, definite history of chronic kidney disease or liver disease, and pre-existing blood purification.

### 2.2 CRRT protocol

CRRT was applied to critical ill patients with SA-AKI at a rate of 30–35 mL/kg/h in continuous veno-venous hemofiltration (CVVH) mode. The CRRT was delivered using PRISMAFLEX machine equipped with M100 (surface area, 1.0 m^2^) filter set, which is composed of hollow fibers made of acrylonitrile and sodium methallyl sulfonate copolymer AN69 HF (PRISMAFLEX). The vascular access was obtained by inserting a 14F double-lumen catheter into the femoral vein. Additionally, the transmembrane pressure (TMP) was closely monitored to ensure that it remained between 100 and 300 mmHg. The composition of the replacement solution is tailored to the patient’s electrolyte levels. Regional citrate anticoagulation was used in all patients underwent CRRT. Monitor both Total Calcium (Total Ca) and ionized Calcium (iCa) levels before and after the filter, and adjust the rate of citrate infusion dynamically to maintain Total Ca levels between 2.2 and 2.7 mmol/L, post-filter iCa levels between 0.2 and 0.4 mmol/L, and pre-filter iCa levels between 1.0 and 1.2 mmol/L. Ensure the ratio of total Ca/iCa ≤2.1. These approaches help to keep the filter patent, reduces the risk of bleeding, and prevents the accumulation of citrate.

### 2.3 Clinical and biochemical data collection

Upon sighing the informed consent, the demographic, clinical and biochemical data of patients during their stay in ICU were obtained by specialized and fixed interviewers. Traditional prognostic indexes including the Acute Physiology and Chronic Health Evaluation II (APACHE II) and the Sequential Organ Failure Assessment (SOFA) scores were also recorded. APACHE II scores range from 0 to 67, while SOFA scores range from 0 to 24. The higher the score of any scale, the more severe the illness and the greater the risk of death.

### 2.4 Blood sample collection and storage

At the beginning (T0) and 72 h (T72) of CRRT, blood samples were collected in ethylenediaminetetraacetic acid (EDTA) tubes and placed at 4°C, and then centrifuged at 3,000 rpm for 10 min at 4°C within 8 h by professional experimenters to obtain plasma aliquots (100 μL), which were packed into numbered EP tubes (on ice) and stored at −80°C. After all the plasma samples are collected, they were detected and analyzed in the same batch to avoid differences between batches. All procedures are operated in dark to avoid decomposition or further reaction of oxidized lipids.

### 2.5 Oxidative lipidomics analysis

#### 2.5.1 Chemicals and reagents

All eicosanoids and deuterated internal standards were purchased from Cayman Chemical. HPLC-grade acetonitrile and methanol were obtained from Merck (Darmstadt, Germany). MilliQ water (Millipore, Bradford, United States) was used in all experiments. Acetic acid was purchased from Sigma-Aldrich. CNW Poly-Sery MAX SPE cartridges were from ANPEL Co. (Shanghai, PRC). The stock solutions of standards were prepared by dissolving them in methanol at a concentration of 5 μg/mL. All stock solutions were stored at −80°C and diluted with methanol to create working solutions prior to analysis.

#### 2.5.2 Sample preparation and extraction

All the samples were thawed on the ice. A 200 μL methanol/acetonitrile (1:1, v/v) solution containing internal standard were added into the 100 μL sample and vortexed for 5 min. After the vortexing step, the mixture was then subjected to protein precipitation at a low temperature of −20°C for 30 min. This step helps to remove proteins from the samples. The samples were centrifuged at 12,000 rpm for 10 min (4°C). The entire supernatant was carefully collected and transferred into a new tube. To ensure thorough extraction, the process was repeated once more, and the supernatants from both extractions were combined. To extract the eicosanoids from the supernatants, Poly-Sery MAX solid-phase extraction (SPE) columns (ANPEL) were used. Prior to analysis, the eluent was dried under vacuum and redissolved in 100 μL of a methanol/water (1:1, v/v) mixture for UPLC/MS/MS analysis.

#### 2.5.3 UPLC conditions

The sample extracts were analyzed using a LC-ESI-MS/MS system (UPLC, ExionLC AD, https://sciex.com.cn/; MS, QTRAP® 6,500+ System, https://sciex.com/). The analytical conditions were as follows, HPLC: column, Waters ACQUITY UPLC HSS T3 C18 (100 mm × 2.1 mm i.d., 1.8 µm); solvent system, water with 0.04% acetic acid (A), acetonitrile with 0.04% acetic acid (B); The gradient was 0–2.0 min from 0.1% to 30%B; 2.0–4.0 min to 50% B; 4.0–5.5 min to 99% B, which was maintained for 1.5 min; and 6.0–7.0 min reduced to 0.1% B and maintained for 3.0 min flow rate, 0.4 mL/min; temperature, 40°C; injection volume: 10 μL.

#### 2.5.4 ESI-MS/MS conditions

Linear ion trap and triple quadrupole scans were acquired on a triple quadrupole-linear ion trap mass spectrometer (QTRAP), QTRAP® 6,500+ LC-MS/MS System, equipped with an ESI Turbo Ion-Spray interface, operating in negative ion mode and controlled by Analyst 1.6.3 software (Sciex). The ESI source operation parameters were as follows: ion source, ESI-; source temperature 550°C; ion spray voltage (IS) −4500 V; curtain gas (CUR) was set at 35 psi, respectively. Eicosanoids were analyzed using scheduled multiple reaction monitoring (MRM). Data acquisitions were performed using Analyst 1.6.3 software (Sciex). Multiquant 3.0.3 software (Sciex) was used to quantify all metabolites. Mass spectrometer parameters including the declustering potentials (DP) and collision energies (CE) for individual MRM transitions were done with further DP and CE optimization. A specific set of MRM transitions were monitored for each period according to the metabolites eluted within this period.

### 2.6 Quality control analysis

The Quality control (QC) samples, consisting of a mixture of all the samples, were included in the queue for analysis, with one QC sample inserted after every 10 test samples. The total ion current (TIC) chromatogram of the same QC sample was displayed by overlapping, and the separation trend analysis in the principal component analysis (PCA) model was analyzed to monitor the stability of the instrument. Additionally, Pearson correlation analysis was conducted to ensure the stability of the detection process.

In omics research, the ratio of the standard deviation to the mean of the raw data, known as the Coefficient of Variation (CV), is commonly used to reflect the degree of data dispersion. By analyzing the CV values of QC samples using the Empirical Cumulative Distribution Function (ECDF), we can assess the variability of the data. A higher proportion of substances with low CV values in QC samples indicates that the experimental data is more stable.

### 2.7 Lipid species and raw data processing

A total of 141 downstream metabolites derived from the oxidation of various fatty acids including arachidonic acids (AA), linoleic acid (LA), α-linolenic acid (ALA), docosahexaenoic acid (DHA), eicosapentaenoic acid (EPA) and dihomo-gamma (γ)- linolenic acid (DGLA) were quantified. The mass spectrum data was processed using Analyst 1.6.3 software. Standard substances with different concentration gradients were prepared, and the standard curves for each substance were constructed. The concentration ratio of the external standard to the internal standard was used as the horizontal coordinate, while the area ratio was used as the vertical coordinate. Then calculate the corresponding sample content based on the standard curves.

### 2.8 Statistical analysis

Demographic and baseline clinical characteristics analysis were performed using SPSS27.0. Depending on the distribution normality determined by Kolmogorov-Smirnov test, continuous variables were summarized as mean and standard deviation (SD) or median and inter-quartile range (IQR) through the Student *t*-test (two tailed) or Mann–Whitney U-test respectively. Differences between groups of categorical variables were determined by fisher’s exact test or continuous correction chi-square test.

Unsupervised analysis principal component analysis (PCA) and supervised analysis Orthogonal Projections to Latent Structures Discriminant Analysis (OPLS-DA) were conducted to evaluate the profiling of metabolites in each group using R package “prcomp”, “MetaboAnalystR” (R version 3.5.1). Significant difference oxidized lipids between survivors and non-survivors were determined by using Variable Importance in Projection (VIP) > 1, P-value <0.05, and fold change (FC) ≥ 2 or ≤0.5.

To further reduce variables, the Least Absolute Shrinkage and Selection Operator (LASSO) and Random Forest (RF) were conducted by using R package “readxl”, “glmnet”, “tidyverse”, “varSelRF” (R version 4.2.2). RF is known as one of the best algorithms for evaluating the role of various variables in classifying data, but it is insensitive to multivariate collinearity. LASSO regression adeptly fits generalized linear models, incorporating both variable selection and complexity control. This approach yields models that are more streamlined, effectively circumventing the statistical challenges posed by multicollinearity.

## 3 Results

### 3.1 Patient characteristics

**FIGURE 1:**
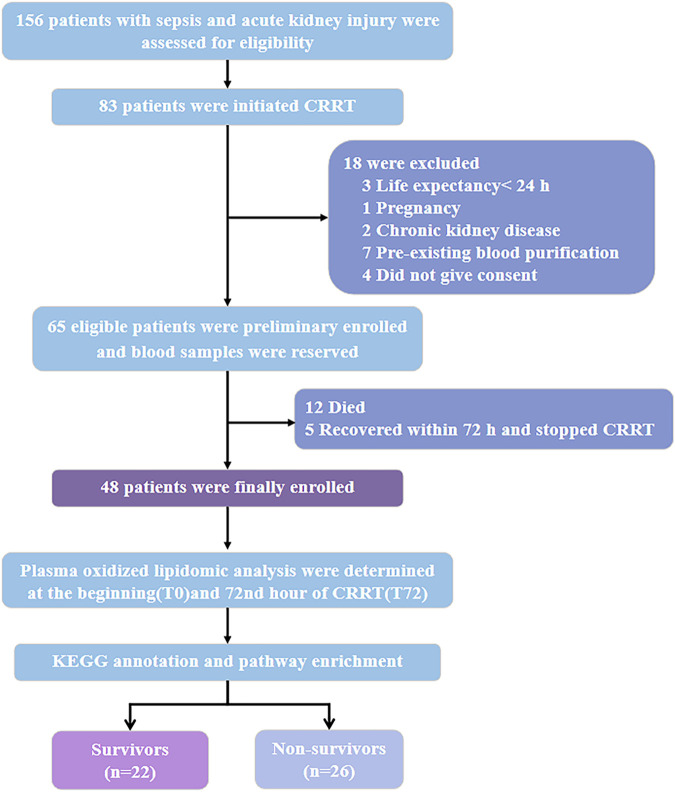
Study design and data analysis workflow. Abbreviations: CRRT, continuous renal replacement therapy; KEGG, Kyoto Encyclopedia of Genes and Genomes.

Of the 65 patients with SA-AKI who received CRRT, 17 patients discontinued CRRT within 72 h because of recovery (5 cases) or death (12 cases) (Figure 1). The remaining 48 patients who received CRRT for more than 72 h were finally enrolled in this study, of which 22 patients were discharged from ICU (Figure 1). The overall mortality of patients with SA-AKI received CRRT for more than 72 was 54.17% (n = 26). 39.58% of the patients had oliguria (19 cases), and had to need mechanical ventilation (17 cases, 35.42%) and vasopressors (20 cases, 41.67%) (Table 1). The majority of the patients were in AKI stage III by KIDGO standard (45 cases, 93.75%) (Table 1). Compared with survivors, non-survivors have higher levels of blood urea nitrogen and bilirubin, and more demand for mechanical ventilation and vasopressor drugs (Table 1). SOFA scores of non-survivors were also significantly higher than that of survivors (Table 1).

**TABLE 1:** Demographic, baseline clinical characteristics of SA-AKI patients.

Variables	All patients (n = 48)	Survivors (n = 22)	Non-survivors (n = 26)	*t*/*χ* ^*2*^/*Z*	P-value
Age (y)^a^	61.5 (50.5.67.8)	62.500 (35.8.70.0)	60.500 (54.5.65.5)	−0.031	0.975
Male sex, n (%)^d^	37 (77.08)	16 (72.73)	21 (80.77)	0.436	0.509
WBC (×10^9^/L)^a^	11.808 (6.90.19.87)	9.689 (6.2.26.8)	12.423 (8.9.18.4)	−0.538	0.591
NEUT%, (%)^c^	89.818 ± 7.683	91.96 ± 8.41	88.00 ± 6.64	1.823	0.075
HGB, (g/L)^a^	96.28 (81.13,118.19)	102.080 (81.6,120.9)	89.725 (80.3,116.4)	−0.859	0.39
PLT (×10^9^/L)^a^	102.46 (50.83,142.42)	121.955 (57.9,140.0)	57.855 (50.3,168.8)	−0.393	0.694
sCr (μmol/L)^a^	213.596 (172.90,354.67)	329.086 (171.4,359.3)	206.208 (173.6,271.4)	−0.869	0.385
BUN (mmol/)^c^	22.724 (15.36.27.74)	19.372 (14.9.24.8)	26.725 (19.6.34.2)	−2.359	0.018*
CO_2_ (mmol/L)^a^	21.385 (18.92.22.9)	21.295 (18.3.22.5)	21.400 (18.1.23.0)	−0.331	0.741
ALB (g/L)^a^	29.38 ± 3.883	29.84 ± 3.91	28.99 ± 3.89	0.744	0.461
AST (U/L)^a^	86.1 (55.08,234.82)	73.185 (43.7,246.8)	93.060 (77.2,167.4)	−0.879	0.379
ALT (U/L) ^a^	49.72 (33.66,205.64)	59.180 (27.6,219.1)	48.880 (45.4.89.4)	−0.186	0.852
TBIL (μmol/L)^a^	28.38 (14.21,255.03)	26.495 (11.4.34.5)	244.199 (14.7,478.1)	−3.021	0.003**
DBIL (μmol/L)^a^	21.474 (7.45,213.74)	12.907 (8.1.31.2)	204.665 (7.2,440.6)	−2.193	0.028*
IBIL (μmol/L)^a^	9.695 (7.00.36.00)	8.623 (3.0.12.2)	35.287 (7.5.40.2)	−3.414	0.001**
PCT (ng/mL)^a^	23.545 (6.95.40.19)	24.544 (12.0.45.1)	22.903 (6.2.39.4)	−1.076	0.282
SA-AKI stage III, n (%)^b^	45 (93.75)	19 (86.36)	26 (100.00)	3.782	0.052
APACHE II^a^	20.729 ± 8.22	19.82 ± 10.71	21.50 ± 5.42	−0.668	0.509
SOFA^a^	10 (8.00.16.25)	8.500 (7.0.11.3)	12.000 (9.8.18.0)	−3.104	0.002**
Mechanical ventilation, n (%)^d^	17 (35.42)	2 (9.09)	15 (57.69)	12.306	0.000**
Need of vasopressor, n (%)^d^	20 (41.67)	5 (22.73)	15 (57.69)	5.994	0.014*
Oliguria, n (%)^d^	19 (39.58)	9 (40.91)	10 (38.46)	0.03	0.863

Abbreviations: WBC, white blood cell; NEUT%, neutrophil%; HGB, haemoglobin; PLT, platelet; sCr, serum creatinine; BUN, urea nitrogen; ALB, albumin; ALT, alanine aminotransferase; AST, aspartate aminotransferase; ALT, glutamic-pyruvic transaminase; TBIL, total bilirubin; DBIL, Direct bilirubin; IBIL, Indirect bilirubin; PCT, procalcitonin; APACHE II Score, Acute Physiology and Chronic Health Evaluation II, Score; SOFA, Score, Sequential Organ Failure Assessment.

^a^ Values are expressed as median (interquartile range) (Mann–Whitney U-test).

^b^ Values are expressed as number (percentage) (Fisher’s exact test).

^c^ Values are expressed as mean ± standard deviation (Student’s t-test).

^d^ Values are expressed as number (percentage) (continuous correction chi-square test).

*P < 0.05, **P < 0.01.

### 3.2 Plasma oxidative lipidomics profiling

To investigate the underlying relationship among multiple variables, we utilized the prcomp function in the R software to normalize the data. Following the removal of unidentified substances and interferon ion pairs through UPLC-MS/MS, 124 metabolites were successfull identified. The TIC chromatogram of the QC (quality controls) samples showed that the retention time is consistent with the peak intensity, and the curves are highly coincident, indicating that the signals were stable throughout the analysis process (Supplementary Figure S1A). The PCA plot showed that QC samples were highly clustered (Supplementary Figure S2B), and the empirical cumulative distribution function (ECDF) showed that the coefficient of variation (CV) value of more than 80% substances was less than 0.2 (Supplementary Figure S3C). The above evidences proved that the QCs had good consistency with the tested samples in the quantification of plasma oxidized lipids.

### 3.3 Characteristics of plasma oxidized lipids in non-survivors versus survivors at T0

**FIGURE 2:**
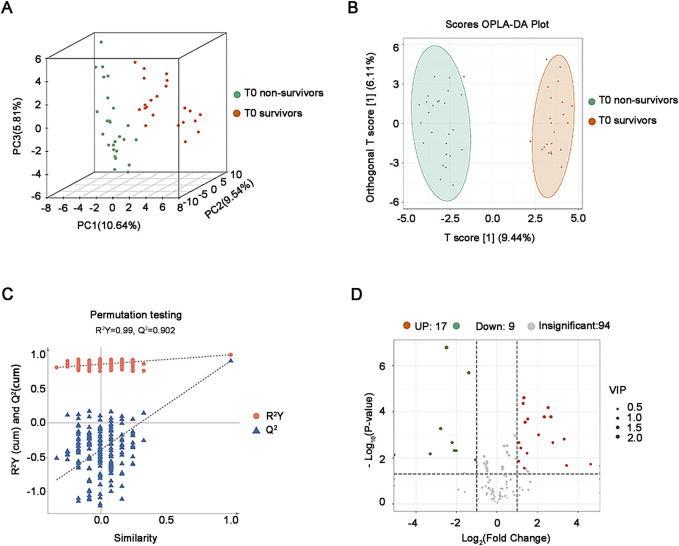
Analysis of plasma oxidative lipidomics in critical SA-AKI patients at T0**. (A)** 3D PCA of survivors and non-survivors at T0. **(B)** OPLS-DA Score plot of survivors and non-survivors at T0. **(C)** Permutation testing of the OPLS-DA model in critical SA-AKI patients at T0. Q^2^ represents predictive and R^2^Y represents interpretability of the OPLS-DA model. Q^2^ = 0.902, R^2^Y = 0.99. **(D)** Volcano plots of all oxidized lipids in non-survivors and survivors at T0. Abbreviations: PCA, principal component analysis; OPLS-DA, orthogonal projections to latent structures discriminant analysis.

The results of both PCA (Figure 2A) and OPLS-DA plot (Figure 2B) showed that the plasma oxidized lipids were significantly separated between non-survivors and survivors. Permutation testing was used to verify the stability and reliability of the OPLS-DA model and avoid over-fitting. Results show that the predictive ability and interpretability of the OPLS-DA model was outstanding with R^2^Y = 0.99, Q^2^ = 0.902 (Figure 2C). According to the criteria of VIP≥1, *P* < 0.05, and FC ≥ 2 or ≤0.5, 26 different oxidized lipids were identified between non-survivors and survivors (Figure 2D).

**FIGURE 3:**
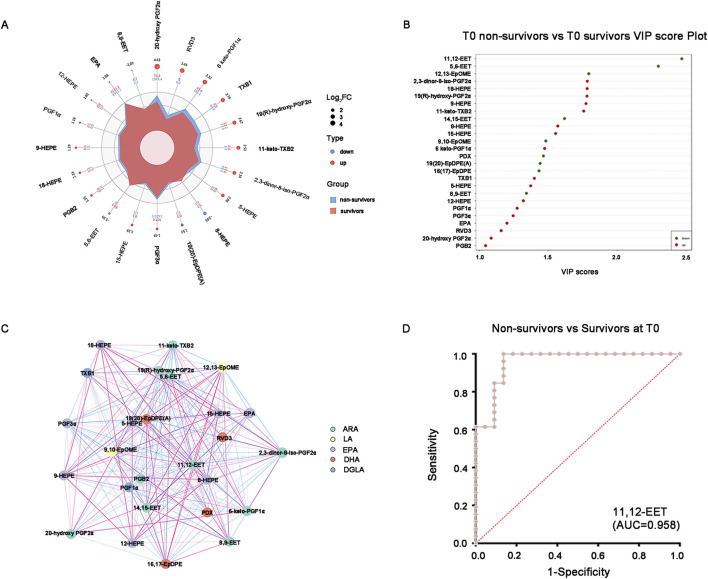
Promising oxidized lipids for distinguishing survivors and non-survivors at T0. **(A)** Combined radar chart of the different oxidized lipids between survivors and non-survivors at T0. The outermost layer represents the name of the substance. The second layer displays the log_2_FC value of the substance between the two groups. The third layer represents the absolute value of the log_2_FC of substances in the two groups using point size. The fourth layer indicates the quantitative mean of substances in the two groups. The fifth layer shows the differences in the mean values of substances between the two groups after log2 processing, based on their area size. **(B)** VIP score plot of the different oxidized lipids between survivors and non-survivors at T0. The top 20 differential oxidative lipids with the highest VIP Score were displayed. **(C)** The correlation network diagram of the different oxidized lipids between survivors and non-survivors at T0. The red line indicates a positive correlation, whereas the blue line indicates a negative correlation. The thickness of the line corresponds to the absolute value of the correlation coefficient, with thicker lines indicating stronger correlations. **(D)** ROC curve of the 11,12-EET distinguishes the non-survivors from survivors at T0. AUC = 0.958. Abbreviations: VIP, variable importance in projection; ROC, receiver operating characteristic; AUC, area under the curve.

Among them, the vasodilatory and anti-inflammatory oxidized lipids represented by epoxyeicosatrienoic acids (EETs), epoxyoctadecaenoic acid (EpOMEs) and epoxydocosapentaenoic acids (EpDPEs) were significantly lower in non-survivors than those in survivors according to Radar chart and VIP score plot, while the oxidized lipids involved in vasoconstriction, pro-inflammation and cardiotoxicity such as the prostaglandins (PGs) and thromboxane (TXB) were significantly higher in non-survivors than those in survivors (Figures 3A, B). Unexpected, the hydroxyeicosapentaenoic acids (HEPEs), as important members of specialized pro-resolving mediators (SPMs), were also up-regulated in non-survivors (Figures 3A, B). Pearson correlation analysis is instrumental in facilitating our thorough comprehension of the interconnected regulatory relationship among the variables and displayed by the correlation network diagram vividly (Figure 3C). The results showed that the expression of the EETs were positively correlated with EpOMEs and EpDPEs, but negatively correlated with the PGFs in critical SA-AKI patients at T0 (Figure 3C). To further explore the potential candidates for prediction the prognosis in critical SA-AKI patients, we incorporated the different baseline clinical data and the different oxidized lipids selected through the OPLS-DA model into RF and LASSO analyses. As the only intersecting variable filtered out through RF and LASSO analyses, 11,12-EET was identified as the most promising biomarker with an AUC of 0.958 (Figure 3D). This finding suggests that the down-regulation of 11,12-EET can effectively identify critical ill patients with SA-AKI who are highly likely to meet a poor prognosis.

**FIGURE 4:**
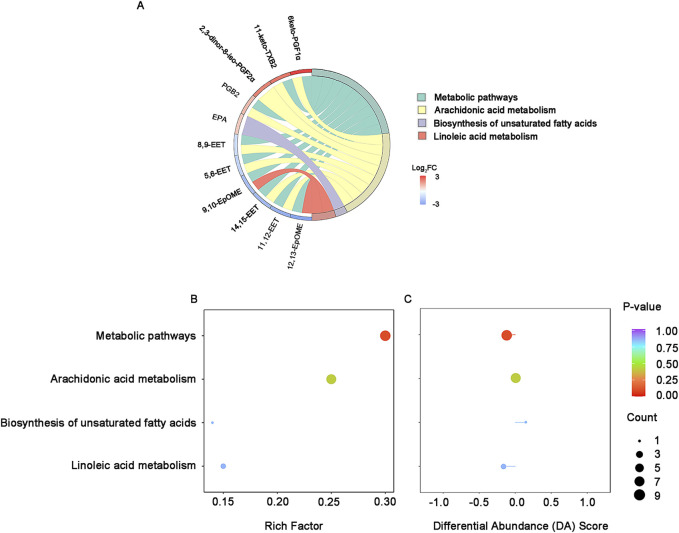
KEGG enrichment and integral analysis of differential oxidized lipids between survivors and non-survivors at T0**. (A)** KEGG enrichment chord diagram at T0. **(B)** KEGG enrichment bubble plots of the differential oxidized lipids in survivors and non-survivors at T0. The color of bubbles represents the value of adjusted p-value and the significance of this pathway. The size of bubbles represents the number of annotated differential oxidative lipids. **(C)** DA Score. The horizontal coordinate reflects the expression trend of all identified metabolites in target pathways, and the ordinate indicates the difference path name. Abbreviations: KEGG, Kyoto Encyclopedia of Genes and Genomes; DA score: differential abundance score.

Of the 26 differential lipids, fourteen were annotated in KEGG database and 11 of them were enriched in arachidonic acid metabolism, metabolic pathways and linoleic acid metabolism (Figure 4A). Among the 4 enriched pathways, the most significant enrichment is the metabolic pathways, which is slightly down-regulated with linoleic acid metabolism, while arachidonic acid metabolism remained unchanged (Figures 4B, C).

**FIGURE 5:**
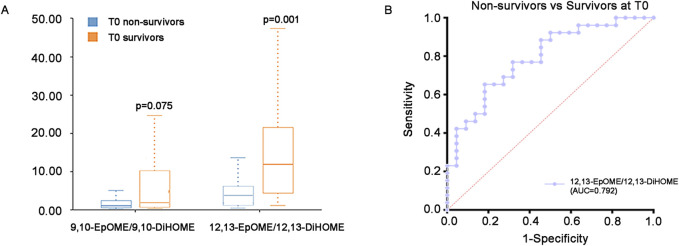
EpOMEs/DiHOMEs ratio at T0. **(A)** Difference of EpOMEs/DiHOMEs ratio between survivors and non-survivors at T0. **(B)** ROC curve of the 12,13-EpOME/12,13-DiHOME between the survivors and non-survivors at T0. AUC = 0.792. Abbreviations: ROC, receiver operating characteristic.

Additionally, in view of the important role of EpOMEs/DiHOMEs in anti-/pro-inflammation and vasodilatation/vasoconstriction, we further analyzed ratio of EpOMEs/DiHOMEs in the survivors and non-survivors at T0. Results have shown that the 12,13-EpOME/12,13-DiHOME were significantly higher in the survivors (P < 0.05), while the difference of 9,10-EpOME/9,10-DiHOME were not statistically significant (Supplementary Table S1) (Figure 5A). ROC curve displayed the strength of 12,13-EpOME/12,13-DiHOME in predicting the prognosis of critical SA-AKI patients with an AUC of 0.792 (Figure 5B).

### 3.4 Specific oxidized lipids alteration during CRRT

To assess the impact of CRRT on the plasma oxidized lipids in SA-AKI patients, we analyzed the data of T72 vs. T0 in survivors and non-survivors respectively. The 3D PCA (Supplementary Figures S2A, D) and OPLS-DA (Supplementary Figures S2B, E) plots showed that the differences of oxidized lipid composition before and after CRRT were significant in both non-survivors and survivors. The permutation testing indicated that the OPLS-DA models for both survivors and non-survivors were robust and there were no signs of overfitting (Supplementary Figures S2C, F).

**FIGURE 6:**
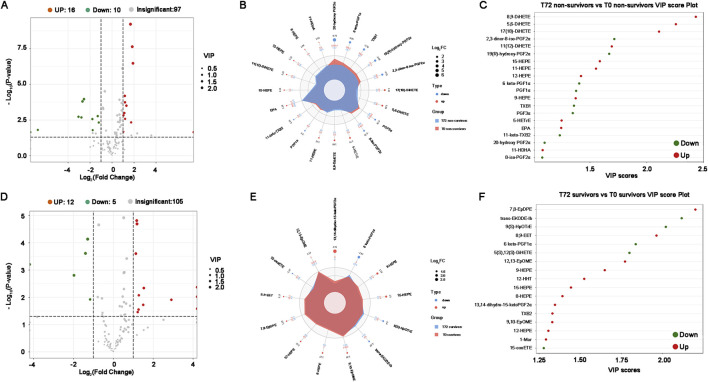
Differential oxidized lipids between T0 and T72 in critical SA-AKI patients undergoing CRRT**. (A)** Volcano plots of all oxidized lipids between T0 and T72 in non-survivors. **(B)** Combined radar chart of the different oxidized lipids between T0 and T72 in non-survivors. **(C)** VIP score plot of the differential oxidized lipids between T0 and T72 in non-survivors. **(D)** Volcano plots of all oxidized lipids between T0 and T72 in survivors. **(E)** Combined radar chart of the different oxidized lipids between T0 and T72 in survivors. **(F)** VIP score plot of the differential oxidized lipids between T0 and T72 in survivors. Abbreviations: VIP, Variable Importance in Projection.

The volcano plot validates that following over 72 h of CRRT treatment, a total of 26 kinds of oxidized lipids exhibited significant alterations in non-survivors, with 16 being up-regulated and 10 being down-regulated (Figure 6A). Compared to T0, DiHETEs in non-survivors at T72 exhibited the most significant upregulation with the highest VIP score, while the PGFs were the primary down-regulated species and contributed the largest FC score (Figures 6B, C). Survivors who underwent CRRT treatment for more than 72 h displayed a total of 17 different oxidized lipids before and after treatment (Figure 6D). In comparison to T0, HEPEs, EpOMEs, and 7,8-EpDPE were all up-regulated in the survivors at T72, with 7,8-EpDPE being the most notably elevated (Figures 6E, F).

**FIGURE 7:**
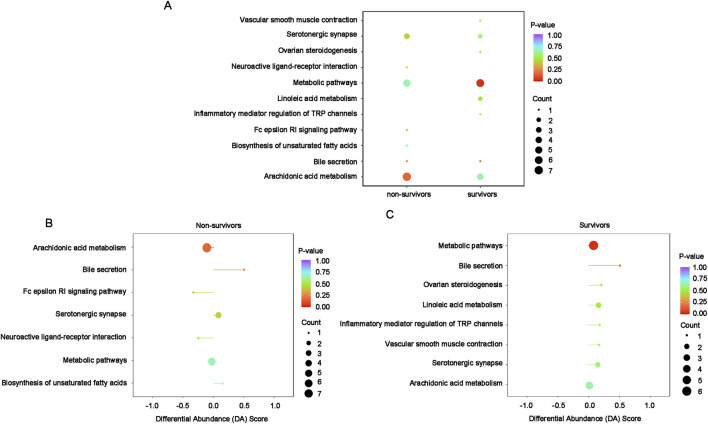
KEGG enrichment and integral analysis of differential oxidized lipids between T0 and T72 in survivors and non-survivors**. (A)** Combination enrichment bubble chart shows the enrichment pathways of differential lipids in survivors and non-survivors. The color of bubbles represents the value of adjusted p-value and the significance of this pathway. The size of bubbles represents the number of annotated differential oxidative lipids. **(B)** DA Score of the enriched pathways in non-survivors between T0 and T72. The horizontal coordinate reflects the expression trend of all identified metabolites in target pathways, and the ordinate indicates the difference path name. **(C)** DA Score of the enriched pathways in survivors between T0 and T72.

The KEGG annotation and enrichment analysis indicate that differentially oxidized lipids are mainly enriched in arachidonic acid metabolism among non-survivors, although the enrichment is not significant, while significantly enriched in metabolic pathways among survivors (Figure 7A). In non-survivors, the main enriched pathway, arachidonic acid metabolism, showed a slight downregulation (Figure 7B). In survivors, the significantly enriched pathway, metabolic pathways, exhibited an overall slight upregulation (Figure 7C).

The ratio of EpOMEs/DiHOMEs between T72 and T0 were compared in survivors and non-survivors, respectively. Results have shown that the ratio of 9,10-EpOME/9,10-DiHOME and 12,13-EpOME/12,13-DiHOME were both significantly up-regulated in survivors at T72, while the alteration were not significant in the non-survivors (Table 2).

**TABLE 2:** Ratio of EpOMEs/DiHOMEs in non-survivors and survivors at T0 and T72.

Variables	Non-survivors				survivors			
	T0 (n = 26)	T72 (n = 26)	Z	P-value	T0 (n = 22)	T72 (n = 22)	Z	P-value
9,10-EpOME/9,10-DiHOME	0.972 (0.5.2.3)	1.141 (0.6.1.8)	0.018	0.985	1.794 (0.7.10.2)	9.214 (2.7.15.1)	−2.136	0.033*
12,13-EpOME/12,13-DiHOME	3.548 (1.1.6.0)	2.598 (1.7.3.9)	0.549	0.583	11.799 (4.3.21.5)	35.001 (24.3.53.8)	−3.896	0.000**

*P < 0.05, **P < 0.01.

## 4 Discussion

CRRT is widely employed for delivering renal support to critically ill patients suffering from AKI, especially those who are experiencing hemodynamic instability. Modes, doses and initiation timing for CRRT are individualized set to correcting water, electrolyte and acid-base imbalance, stabilizing internal environment and eliminating inflammatory mediators. However, many doctors and nephrologists in ICU are still faced with the predicament of lacking clinically available tools for identify critical ill patients with SA-AKI who can benefit from CRRT more than its adverse complications. Furthermore, it is still unclear whether alterations in plasma solutes induced by CRRT have an impact on the immune microenvironment and whether this effect is associated with patient prognosis. In this study, oxidative lipidomics was used for the first time to explore the specific oxidized lipids closely associated with the prognosis of critical ill patients with SA-AKI undergoing CRRT and then explore the new mechanism of CRRT. The main highlights were displayed as following. First, at T0, compared with that in survivors, plasma EETs, EpOMEs and EpDPEs in non-survivors were down-regulated, while plasma PGFs, TXB and HEPEs were up-regulated, suggesting that the imbalance of vasoconstriction/vasodilatation and pro-inflammatory/anti-inflammatory mediators were closely associated with the prognosis of critical ill patients with SA-AKI. Second, after 72 h of CRRT, DiHETEs and HEPEs in plasma of non-survivors increased and PGFs decreased, while HEPEs, EpOMEs and 7,8-EpDPE in plasma of survivors increased and 6keto-PGF1α decreased, suggesting that CRRT may reduce pro-inflammatory response and vasoconstriction by clearing PGFs and promote the production of anti-inflammatory HEPEs by activating EPA metabolism. Third, for critical ill patients with SA-AKI who are about to undergo or already undergoing CRRT, it may be necessary to adjust CRRT prescription, strengthen anti-infection and supplement vasodilators in time to improve the prognosis if the EETs, EpDPEs, EpOMEs, and HEPEs persistent low levels or DiHETEs persistent high levels.

Oxidized lipids are the metabolites of polyunsaturated fatty acids (PUFAs) triggered by enzymatic or non-enzymatic reactions. Among the enzymatic reactions, PUFAs undergo oxygenation through the action of several enzymes, such as cyclooxygenases (COXs), lipoxygenases (LOXs), CYP 450-epoxygenases, and ω-hydroxylases, resulting in the formation of various metabolites (Hanif et al., 2017a; Hanif et al., 2017b; Hanif et al., 2017c). The CYP 450 is the latest emerged but not the least important pathway, which is a member of the membrane-bound enzymes families located in liver, brain, kidneys, lung, heart, and the cardiovascular system. One of the crucial physiological functions of CYP450-epoxygenases is the conversion of arachidonic acid (AA) and linoleic acid (LA) into epoxyeicosatrienoic acids (EETs) and epoxyoctadecaenoic acid (EpOMEs), which can hyperpolarize vascular smooth muscle (VSM) cells by activating the large-conductance calcium-activated potassium (BK) channels in cyclic adenosine monophosphate (cAMP) and protein kinase A-dependent mechanisms, acting as potent vasodilators in renal arterioles (Imig et al., 1996; Roman, 2002; Fijałkowski et al., 2018). Previous studys have demonstrated that EpOMEs can protect renal proximal tubular cells from hypoxia/reoxygenation injury (Konkel and Schunck, 2011). Besides, EETs have vascular relaxation, anti-inflammatory and fibrinolytic activities by activating KATP channels, p42/p44 MAPK, and protein kinase A signaling, as well as inhibiting NF-κB (Hanif et al., 2016a; Hanif et al., 2016b; Yadav et al., 2016; Hanif et al., 2017a; Hanif et al., 2017b; Hanif et al., 2017c). EpDPEs is also a noteworthy class of CYP450 epoxygenase-catalyzed metabolites, which derived from DHA and can generate vasodilatation by activating the large-conductance BK channels in VSM, with the same mechanism as EETs (Wang et al., 2011; Yang et al., 2022). The persistence of these CYP 450-epoxygenase product deficiencies may lead to disorder of renal blood flow regulation and impaired kidney function. This study showed that the plasma EETs, EpDPEs and EpOMEs in non-survivors were significantly lower than those in the survivors at T0, and machine-learning demonstrated that 11,12-EET can serve as robust predictors of the prognosis of SA-AKI patients undergoing CRRT, suggesting that the death of SA-AKI patients was closely related to the deficiency of these vasodilatory/anti-inflammatory lipids. Notably, CRRT with a duration of not less than 72 h increased the plasma levels of 7,8-EpDPE, 8,9-EET and EpOMEs in survivors, but it was ineffective for non-survivors, suggesting that CRRT seems to improve renal blood flow and promote renal function recovery by increasing oxidized lipids with vasodilatory effect.

The EETs were unstable with a relatively short half-life ranging from 7.9 to 12.3 min (Catella et al., 1990). Contrary to CYP450-epoxygenases, soluble epoxide hydrolase (sEH) can convert EETs into diols or less active metabolites dihydroxyeicosatrienoic acids (DHETs) and EpOMEs into dihydroxyoctadecaenoic acids (DiHOMEs), thus reversing the beneficial effects of epoxy-fatty acids and promoting vasoconstriction and pro-inflammation responses (Hanif et al., 2016a; Hanif et al., 2017a; Nayeem, 2018; Nayeem et al., 2022). Our previous study had demonstrated the ratio of plasma EpOMEs/DiHOMEs were significantly down-regulated in SA-AKI patients than healthy controls (Zhou et al., 2023). This study further confirmed that at T0, the ratio of plasma 12,13-EpOME/12,13-DiHOME in non-survivors was lower than that in survivors. In survivors, the down-regulation of EpOMEs/DiHOMEs were significantly improved by more than 72 h CRRT, while the down-regulation persists during the whole course of CRRT in non-survivors. Above evidence suggests that the generation and metabolism of the vasodilator and anti-inflammatory lipid mediators dominated by EETs, EpOMEs and EpDPEs are crutial to the prognosis in SA-AKI patients, and shed light on the potential mechanism of CRRT in the treatment of SA-AKI patients-promoting the generation of EETs, EpOMEs and EpDPEs and inhibit their transformation into pro-inflammatory and vasoconstrictor mediators. For SA-AKI patients with low ratio of plasma EpOMEs/DiHOMEs and persistent during CRRT, additional supplement of the EETs, EpOMEs and EpDPEs and targets inhibition of the sHE in time may be necessary to improve the prognosis.

Main prostaglandins (PGs) and thromboxanes (TXs) are derived from AA by the action of various COX isoforms (Gabbs et al., 2015; Hanif et al., 2017a; Nayeem, 2018). Except prostaglandin E2(PGE2) and prostaglandin I2 (PGI2), almost all prostaglandins have proinflammatory and vasoconstrictive characteristics, which are strongly associated with cardiovascular events and adverse outcomes in critically ill patients (Sha et al., 2012). 6-keto-PGF1α and PGF2α were associated with cardiac dysfunction and cardiovascular events (Caligiuri et al., 2017; Gunter et al., 2017; Hanif et al., 2017a). 6-keto-PGF1α was also confirmed to increase in ischemia/reperfusion-induced AKI (Mizutani et al., 2009). AA can generate TXA2 via the action of thromboxane synthase (Bauer et al., 2014), which has a half-life of only about 30 s and is then spontaneously converted to thromboxane B2 (TXB2) (Zhou et al., 2015). TXA2 has been proved to induce vasoconstriction and platelet aggregation, while TXB2 is associated with cardiovascular dysfunction (Caligiuri et al., 2016; Caligiuri et al., 2017). In this study, it was shown that CRRT lasting at least 72 h significantly reduced the levels of major plasma PGFs and TXs in both survivors and non-survivors, revealing a new mechanism of CRRT in treating patients with SA-AKI: eliminating PGFs and TXs. Whether a higher dose of CRRT will enhance this effect and bring benefits remains to be further studied.

Dihydroxyeicosatetraenoic acids (DiHETEs), another series metabolites derived from AA via LOX pathways (Wang et al., 2019), ultimately contribute to the generation of extra-platelet leukotriene A4 (LTA4) (Serhan and Sheppard, 1990; Fiore et al., 1992). However, LTA4 is unstable and rapidly hydrolyzed to LTB4 by leukotriene A4 hydrolase (LTA4H) or converted into LTC4 by leukotriene C4 synthase (Vo et al., 2018). Several studies have demonstrated that Leukotriene A4 hydrolase inhibition prevents endothelial injury and modulates the inflammation in acute infection (Tian et al., 2013; Akthar et al., 2015). Additionally, the LOX and leukotriene signaling pathways have been preliminary confirmed to be involved in cisplatin-mediated renal toxicity (Alkhamees et al., 2017; Deng et al., 2017), however, whether it is related to SA-AKI remains undiscovered. In this study, after CRRT lasting at least 72 h, plasma DiHETEs increased in non-survivors but remained unchanged in survivors, suggesting that it may be necessary to adjust CRRT prescription to achieve better removal of DiHETEs.

Most hydroxyeicosapentaenoic acids (HEPEs) are generated from eicosapentaenoic acid (EPA) through lipoxygenases (LO) catalysis, which plays an important role in improving insulin resistance, hypertriglyceridemia and inflammation (Shaikh et al., 2022). It is relatively clear that 18-HEPE is generated from EPA catalyzed by cytochrome P450 monooxygenase (CYP450) or acetylated cyclooxygenase-2 (COX-2), and as an instantaneous intermediate, it is rapidly converted into resolvin E1 (RvE1), which plays a role in improving hepatic steatosis and insulin resistance, enhancing phagocytosis of dying cells, and reducing the recruitment of immune cells secreting pro-inflammatory cytokines (Serhan and Levy, 2018; Shaikh et al., 2022; Bäck, 2023). The remarkable increase of 5-HEPE, 12-HEPE, 15-HEPE and 18-HEPE in liver homogenate were confirmed in the preterm sepsis mouse model induced by caecal serofluid prepared from adult caeca (Ashina et al., 2020). In this study, compared with before CRRT, CRRT for 72 h significantly increased the plasma levels of HEPEs in both survivors and non-survivors, suggesting that CRRT may promote the production of HEPEs with anti-inflammatory effects by activating EPA metabolism, although its mechanism needs further study. In addition, compared with non-survivors, the HEPEs were more significantly up-regulated in survivors after 72 h of CRRT. These suggest that the HEPEs may closely related to the progression and prognosis of SA-AKI patients. CRRT may resolve the inflammation by upregulation of HEPEs, reducing the organ injury including kidney caused by sepsis. Besides, it may be effective to adjust the dose and mode of CRRT and strengthen anti-infection and supportive treatment strategies during CRRT to improve the prognosis of SA-AKI patients. Of course, further evaluation is need for targeted research.

## 5 Limitation

Despite this study pioneered the application of oxidative lipidomics to evaluate the effect of CRRT on the plasma oxidized lipids microenvironment in critical ill patients with SA-AKI, there are still some limitations. First, for ethical reasons, almost all patients with severe SA-AKI have received CRRT, so it is impossible to obtain the plasma oxidative lipidomics data of positive control patients who did not receive CRRT at T72. Second, due to the small number of patients, it is impossible to set up subgroups to distinguish the oxidative lipidomics profile in patients with SA-AKI caused by different pathogens, but at least it provides a basis for further research in a larger cohort. Third, as a ground breaking research, although the new mechanisms and underlying targets of CRRT in the treatment of SA-AKI are put forward, detailed clinical and basic validation studies is still needed to elucidate the lipid metabolism reprogramming during CRRT. Fourth, because of the intricate and diverse nature of metabolic pathways, the functions of many differential oxidized lipids are still unknown, which needs further exploration and investigation.

## 6 Conclusion

In summary, this study provides a comprehensive overview of plasma oxidized lipids in critically ill patients with SA-AKI in the context of CRRT, and further clarifies the specific oxidized lipids and pathways that are associated with prognosis. Additionally, we found that aside from alleviates volume overload and corrects electrolyte imbalances, CRRT can also improve prognosis by ameliorate the immunological microenvironment through removing the PGFs and TXs while simultaneously the HEPEs. Our findings provide new insights for assessing the prognosis of severe SA-AKI patients and optimizing CRRT prescriptions.

## Data Availability

The data presented in the study are deposited in the Metabolights repository, accession number MTBLS11695, www.ebi.ac.uk/metabolights/MTBLS11695.
