# Supplementary material for: Unveiling the immunomodulator role of plasma oxidized lipids in SA-AKI progression: a CRRT perspective
Source: Front Physiol. 2024 Dec 23;15:1412235. doi: 10.3389/fphys.2024.1412235 (PMC11701052; doi:10.3389/fphys.2024.1412235)
Supplement: Supplementary file 1 [file DataSheet1.docx]

Supplementary Material


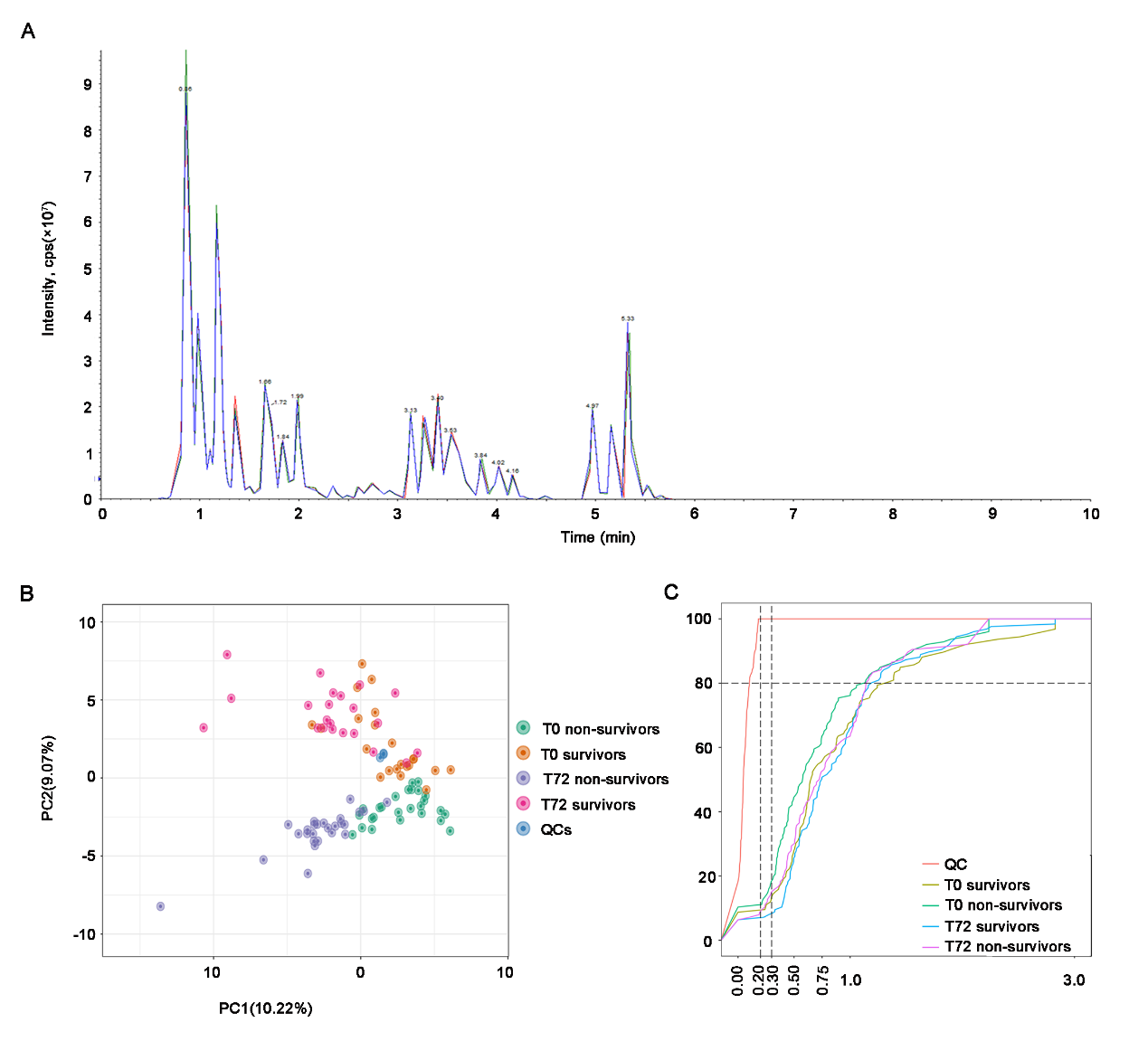


**Supplementary Figure 1.** Plasma Oxidative lipidomics profiling. (A) Overlap display the TIC of QC samples. (B) PCA plot of all samples and QCs. (C) ECDF of all samples and QCs.

Abbreviations: TIC, total ion current graph; QC, quality control; PCA, principal component analysis; QC, quality control sample; ECDF, empirical cumulative distribution function.


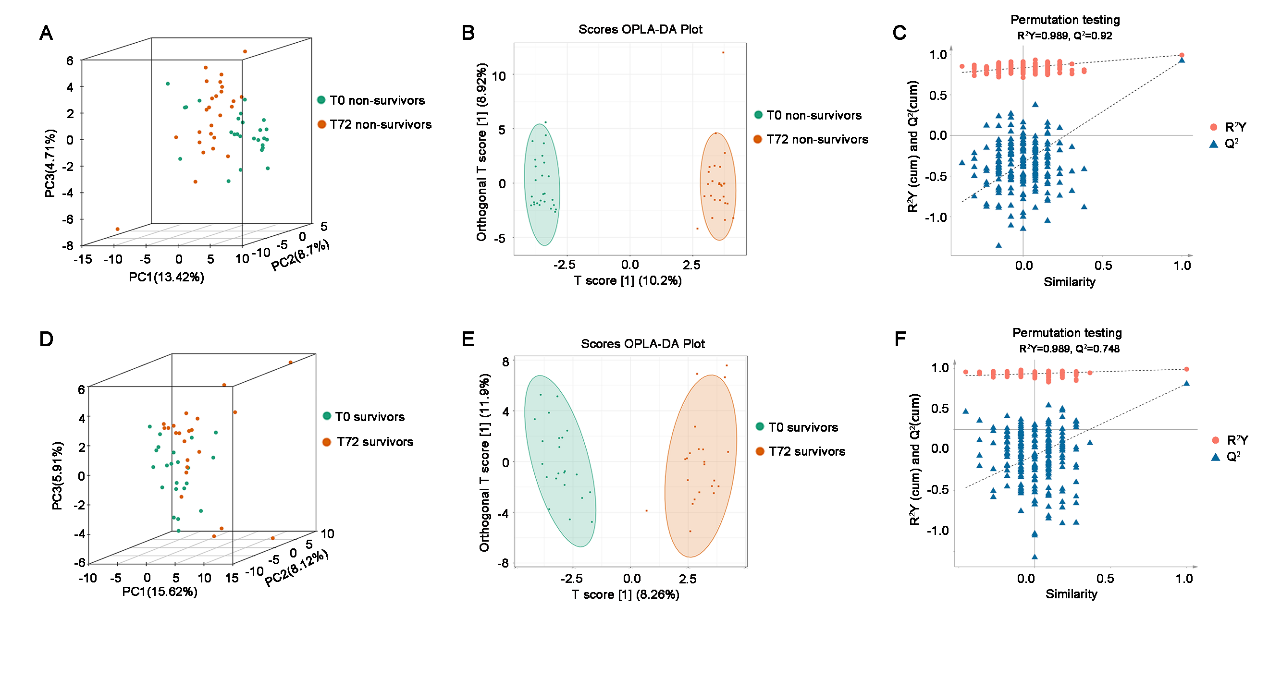


**Supplementary Figure 2.** Analysis of plasma oxidative lipidomics between T0 and T72 in critical SA-AKI patients undergoing CRRT. (A) 3D PCA plot of T0 and T72 in non-survivors. (B) OPLS-DA Score plot of T0 and T72 in non-survivors. (C) Permutation testing of the OPLS-DA model of T0 and T72 in non-survivors. R^2^Y=0.989, Q^2^=0.92. (D) 3D PCA plot of T0 and T72 in survivors. (E) OPLS-DA Score plot of T0 and T72 in survivors. (F) Permutation testing of the OPLS-DA model of T0 and T72 in survivors. R^2^Y=0.989, Q^2^=0.748.

Abbreviations: SA-AKI, sepsis associated acute kidney injury; CRRT, continuous renal replacement therapy; PCA, principal component analysis; OPLS-DA, orthogonal projections to latent structures discriminant analysis.

**Supplementary Table 1. Ratio of** **EpOMEs/DiHOMEs in SA-AKI patients at T0**

| Variables | T0-nonsurvivors  (n=26) | T0-survivors  (n=22) | Z | P value |
| --- | --- | --- | --- | --- |
| 9, 10-EpOME/9, 10-DiHOME | 0.972(0.5,2.3) | 1.794(0.7,10.2) | -1.779 | 0.075 |
| 12, 13-EpOME/12,13-DiHOME | 3.548(1.1,6.0) | 11.799(4.3,21.5) | -3.456 | 0.001** |

*P < 0.05, **P < 0.01
